# Supplementary material for: A Critical View of the Application of the APEX Software (Aqueous Photochemistry of Environmentally-Occurring Xenobiotics) to Predict Photoreaction Kinetics in Surface Freshwaters
Source: Molecules. 2019 Dec 18;25(1):9. doi: 10.3390/molecules25010009 (PMC7017383; doi:10.3390/molecules25010009)
Supplement: Supplementary file 1 [file molecules-25-00009-s001.zip › 3.2.4_gcc-4.4.0.zip!/3.2.4_gcc-4.4.0/3.2.4_gcc-4.4.0/doc/octave/html/interpreter/Random-Number-Generation.html]

Random Number Generation - Untitled


Previous: Distributions,
Up: Statistics


---

### 25.7 Random Number Generation

Octave can generate random numbers from a large number of distributions.
The random number generators are based on the random number generators
described in Special Utility Matrices.

The following table summarizes the available random number generators
(in alphabetical order).

|  |  |
| --- | --- |
| **Distribution** | **Function** |
| Beta Distribution | `betarnd` |
| Binomial Distribution | `binornd` |
| Cauchy Distribution | `cauchy_rnd` |
| Chi-Square Distribution | `chi2rnd` |
| Univariate Discrete Distribution | `discrete_rnd` |
| Empirical Distribution | `empirical_rnd` |
| Exponential Distribution | `exprnd` |
| F Distribution | `frnd` |
| Gamma Distribution | `gamrnd` |
| Geometric Distribution | `geornd` |
| Hypergeometric Distribution | `hygernd` |
| Laplace Distribution | `laplace_rnd` |
| Logistic Distribution | `logistic_rnd` |
| Log-Normal Distribution | `lognrnd` |
| Pascal Distribution | `nbinrnd` |
| Univariate Normal Distribution | `normrnd` |
| Poisson Distribution | `poissrnd` |
| t (Student) Distribution | `trnd` |
| Univariate Discrete Distribution | `unidrnd` |
| Uniform Distribution | `unifrnd` |
| Weibull Distribution | `wblrnd` |
| Wiener Process | `wienrnd` |

— Function File: **betarnd** (a, b, r, c)  
— Function File: **betarnd** (a, b, sz)  
> Return an r by c or `size (`sz`)` matrix of
> random samples from the Beta distribution with parameters a and
> b. Both a and b must be scalar or of size r
> by c.
>
> If r and c are omitted, the size of the result matrix is
> the common size of a and b.

— Function File: **binornd** (n, p, r, c)  
— Function File: **binornd** (n, p, sz)  
> Return an r by c or a `size (`sz`)` matrix of
> random samples from the binomial distribution with parameters n
> and p. Both n and p must be scalar or of size
> r by c.
>
> If r and c are omitted, the size of the result matrix is
> the common size of n and p.

— Function File: **cauchy\_rnd** (lambda, sigma, r, c)  
— Function File: **cauchy\_rnd** (lambda, sigma, sz)  
> Return an r by c or a `size (`sz`)` matrix of
> random samples from the Cauchy distribution with parameters lambda
> and sigma which must both be scalar or of size r by c.
>
> If r and c are omitted, the size of the result matrix is
> the common size of lambda and sigma.

— Function File: **chi2rnd** (n, r, c)  
— Function File: **chi2rnd** (n, sz)  
> Return an r by c or a `size (`sz`)` matrix of
> random samples from the chisquare distribution with n degrees
> of freedom. n must be a scalar or of size r by c.
>
> If r and c are omitted, the size of the result matrix is
> the size of n.

— Function File: **discrete\_rnd** (n, v, p)  
— Function File: **discrete\_rnd** (v, p, r, c)  
— Function File: **discrete\_rnd** (v, p, sz)  
> Generate a row vector containing a random sample of size n from
> the univariate distribution which assumes the values in v with
> probabilities p. n must be a scalar.
>
> If r and c are given create a matrix with r rows and
> c columns. Or if sz is a vector, create a matrix of size
> sz.

— Function File: **empirical\_rnd** (n, data)  
— Function File: **empirical\_rnd** (data, r, c)  
— Function File: **empirical\_rnd** (data, sz)  
> Generate a bootstrap sample of size n from the empirical
> distribution obtained from the univariate sample data.
>
> If r and c are given create a matrix with r rows and
> c columns. Or if sz is a vector, create a matrix of size
> sz.

— Function File: **exprnd** (lambda, r, c)  
— Function File: **exprnd** (lambda, sz)  
> Return an r by c matrix of random samples from the
> exponential distribution with mean lambda, which must be a
> scalar or of size r by c. Or if sz is a vector,
> create a matrix of size sz.
>
> If r and c are omitted, the size of the result matrix is
> the size of lambda.

— Function File: **frnd** (m, n, r, c)  
— Function File: **frnd** (m, n, sz)  
> Return an r by c matrix of random samples from the F
> distribution with m and n degrees of freedom. Both
> m and n must be scalar or of size r by c.
> If sz is a vector the random samples are in a matrix of
> size sz.
>
> If r and c are omitted, the size of the result matrix is
> the common size of m and n.

— Function File: **gamrnd** (a, b, r, c)  
— Function File: **gamrnd** (a, b, sz)  
> Return an r by c or a `size (`sz`)` matrix of
> random samples from the Gamma distribution with parameters a
> and b. Both a and b must be scalar or of size
> r by c.
>
> If r and c are omitted, the size of the result matrix is
> the common size of a and b.
>
>
>
> **See also:** gamma, gammaln, gammainc, gampdf, gamcdf, gaminv.

— Function File: **geornd** (p, r, c)  
— Function File: **geornd** (p, sz)  
> Return an r by c matrix of random samples from the
> geometric distribution with parameter p, which must be a scalar
> or of size r by c.
>
> If r and c are given create a matrix with r rows and
> c columns. Or if sz is a vector, create a matrix of size
> sz.

— Function File: **hygernd** (t, m, n, r, c)  
— Function File: **hygernd** (t, m, n, sz)  
— Function File: **hygernd** (t, m, n)  
> Return an r by c matrix of random samples from the
> hypergeometric distribution with parameters t, m,
> and n.
>
> The parameters t, m, and n must positive integers
> with m and n not greater than t.
>
> The parameter sz must be scalar or a vector of matrix
> dimensions. If sz is scalar, then a sz by sz
> matrix of random samples is generated.

— Function File: **laplace\_rnd** (r, c)  
— Function File: **laplace\_rnd** (sz);  
> Return an r by c matrix of random numbers from the
> Laplace distribution. Or if sz is a vector, create a matrix of
> sz.

— Function File: **logistic\_rnd** (r, c)  
— Function File: **logistic\_rnd** (sz)  
> Return an r by c matrix of random numbers from the
> logistic distribution. Or if sz is a vector, create a matrix of
> sz.

— Function File: **lognrnd** (mu, sigma, r, c)  
— Function File: **lognrnd** (mu, sigma, sz)  
> Return an r by c matrix of random samples from the
> lognormal distribution with parameters mu and sigma. Both
> mu and sigma must be scalar or of size r by c.
> Or if sz is a vector, create a matrix of size sz.
>
> If r and c are omitted, the size of the result matrix is
> the common size of mu and sigma.

— Function File: **nbinrnd** (n, p, r, c)  
— Function File: **nbinrnd** (n, p, sz)  
> Return an r by c matrix of random samples from the Pascal
> (negative binomial) distribution with parameters n and p.
> Both n and p must be scalar or of size r by c.
>
> If r and c are omitted, the size of the result matrix is
> the common size of n and p. Or if sz is a vector,
> create a matrix of size sz.

— Function File: **normrnd** (m, s, r, c)  
— Function File: **normrnd** (m, s, sz)  
> Return an r by c or `size (`sz`)` matrix of
> random samples from the normal distribution with parameters mean m
> and standard deviation s. Both m and s must be scalar
> or of size r by c.
>
> If r and c are omitted, the size of the result matrix is
> the common size of m and s.

— Function File: **poissrnd** (lambda, r, c)  
> Return an r by c matrix of random samples from the
> Poisson distribution with parameter lambda, which must be a
> scalar or of size r by c.
>
> If r and c are omitted, the size of the result matrix is
> the size of lambda.

— Function File: **trnd** (n, r, c)  
— Function File: **trnd** (n, sz)  
> Return an r by c matrix of random samples from the t
> (Student) distribution with n degrees of freedom. n must
> be a scalar or of size r by c. Or if sz is a
> vector create a matrix of size sz.
>
> If r and c are omitted, the size of the result matrix is
> the size of n.

— Function File: **unidrnd** (mx);  
— Function File: **unidrnd** (mx, v);  
— Function File: **unidrnd** (mx, m, n, ...);  
> Return random values from discrete uniform distribution, with maximum
> value(s) given by the integer mx, which may be a scalar or
> multidimensional array.
>
> If mx is a scalar, the size of the result is specified by
> the vector v, or by the optional arguments m, n,
> .... Otherwise, the size of the result is the same as the size
> of mx.

— Function File: **unifrnd** (a, b, r, c)  
— Function File: **unifrnd** (a, b, sz)  
> Return an r by c or a `size (`sz`)` matrix of
> random samples from the uniform distribution on [a, b].
> Both a and b must be scalar or of size r by c.
>
> If r and c are omitted, the size of the result matrix is
> the common size of a and b.

— Function File: **wblrnd** (scale, shape, r, c)  
— Function File: **wblrnd** (scale, shape, sz)  
> Return an r by c matrix of random samples from the
> Weibull distribution with parameters scale and shape
> which must be scalar or of size r by c. Or if sz
> is a vector return a matrix of size sz.
>
> If r and c are omitted, the size of the result matrix is
> the common size of alpha and sigma.

— Function File: **wienrnd** (t, d, n)  
> Return a simulated realization of the d-dimensional Wiener Process
> on the interval [0, t]. If d is omitted, d = 1 is
> used. The first column of the return matrix contains time, the
> remaining columns contain the Wiener process.
>
> The optional parameter n gives the number of summands used for
> simulating the process over an interval of length 1. If n is
> omitted, n = 1000 is used.
